# Mechanisms of Scaling Effect for Emerging Nanoscale Interconnect Materials

**DOI:** 10.3390/nano12101760

**Published:** 2022-05-21

**Authors:** Kai Zhao, Yuanzhao Hu, Gang Du, Yudi Zhao, Junchen Dong

**Affiliations:** 1 Key Laboratory of the Ministry of Education for Optoelectronic Measurement Technology and Instrument, Beijing Information Science and Technology University, Beijing 100101, China; zhaokai@bistu.edu.cn (K.Z.); zhaoyd@bistu.edu.cn (Y.Z.); 2 Huantian Technology (Kunshan) Electronics Co., Ltd., Kunshan 215300, China; 3 School of Software and Microelectronics, Peking University, Beijing 100871, China; 4 School of Integrated Circuits, Peking University, Beijing 100871, China; gangdu@pku.edu.cn

**Keywords:** Monte Carlo method, scattering mechanism, scaling effect, interconnect, resistivity, grain boundary

## Abstract

The resistivity of Cu interconnects increases rapidly with continuously scaling down due to scatterings, causing a major challenge for future nodes in M0 and M1 layers. Here, A Boltzmann-transport-equation-based Monte Carlo simulator, including all the major scattering mechanisms of interconnects, is developed for the evaluation of electron transport behaviors. Good agreements between our simulation and the experimental results are achieved for Cu, Ru, Co, and W, from bulk down to 10 nm interconnects. The line resistance values of the four materials with the inclusion of liner and barrier thicknesses are calculated in the same footprint for a fair comparison. The impact of high aspect ratio on resistivity is analyzed for promising buried power rail materials, such as Ru and W. Our results show that grain boundary scattering plays the most important role in nano-scale interconnects, followed by surface roughness and plasma excimer scattering. Surface roughness scattering is the origin of the resistivity decrease for high-aspect-ratio conductive rails. In addition, the grain sizes for the technical nodes of different materials are extracted and the impact of grain size on resistivity is analyzed.

## 1. Introduction

Cu has been introduced to replace Al in the back end of line (BEOL) of integrated circuit (IC) fabrication as an interconnect material since 1997. Since then, the aggressive down-scaling of Cu BEOL dimensions has led to exponentially increased resistivity [1], which is referred to as the “size effect” [2]. This effect increases the resistance–capacitance (RC) delay, current-resistance (IR) drop, and power consumption at M0 and M1, and thus deteriorates BEOL’s performance [3]. Many efforts have been devoted to improving the BEOL’s performance, from metallization to the structures’ perspective, respectively [3,4,5,6,7,8,9,10].

A straightforward solution is to replace conventional Cu with new materials to meet the conductivity and reliability requirements. The increasing resistivity derives not only from metallic interconnects but also from their diffusion barriers and adhesion liners [7]. Therefore, alternative materials with less contamination, a larger metal-to-barrier volume ratio, better anti-electromigration properties, and acceptable resistivity are desired to replace Cu at M0 and M1 [1,11,12,13,14,15,16,17,18]. Co [1,11,12,13,14], Ru [1,15,16,17], and W [18] are the three promising candidates to meet the above challenges for their better anti-electromigration properties and lower contamination risks, which makes barrierless metallization possible [11].

Besides metallization optimization, structural innovation is deemed a performance booster as well. A typical attempt is to bury the power rails under shallow trench isolation (STI) and Si substrate, which is referred to as buried power rail (BPR) [8]. For example, a power distribution network (PDN) with BPR may achieve a 20% smaller area at the same technology node [10]. In the cases of front-side with buried power rails (FS-BPR), the power rails usually have larger aspect ratios (ARs) to lower their resistivity due to the limitation in the width direction. Abnormal ARs impact the internal grain size and thus affect the resistivity.

There are several theoretical studies with ab initio approaches to explain the resistivity-raising phenomena. However, treating electron transport as one-dimensional coherent scattering processes is rather computationally extensive for interconnect simulations. Simplifications must be made to make this approach practical. For example, normalized full-band relaxation time approximation for the linearized Boltzmann transport equation (BTE) [19] is used to derive the scattering rate in a density-functional-theory (DFT) calculation for metal resistivity [20]. First-principles predictions can be used to determine the product of the bulk resistivity times the bulk electron mean-free-path without calculating the electron scattering explicitly [21,22]. In this approximation, the metal wire resistivity is analytically predicted from the approximate forms of the classical Fuchs and Sondheimer (FS) [23,24] and Mayadas and Shatzkes (MS) [25,26] models. The aforementioned two approaches are all with less rigorous simplifications either in scattering terms or in the transport process.

In the deca-nanometer region, the drift diffusion (DD) approach no longer meets the accuracy requirements in the state-of-art technology nodes. Solving BTE under a semiclassical frame is the most appropriate way to investigate carrier transport behaviors in semiconductors and metals because it may include various scattering mechanisms explicitly instead of a relaxation time approximation at acceptable computational costs. Unfortunately, unlike the BTE approach, which has been widely applied in semiconductor device simulation, its applications in promising M0, M1, and BPR metallic lines with explicit scattering terms have not been reported yet. In Refs. [27,28], the BTE is solved with a relaxation time approximation to calculate the metallic nanowire resistivity, but the estimations are very rough.

In this paper, the BTE is solved with explicit scattering terms by the Monte Carlo (MC) approach to investigate the electron transport properties in Cu, Ru, Co, and W. Our MC simulator is redeveloped and validated based on our previous work for interconnects [29] and for semiconductors [30]. To extend the simulator down to deca-nanometer range, as well as to include the emerging materials, such as Ru, Co, and W, grain boundary scattering (GBS) and surface roughness scattering (SRS) models are modified and their parameters are calibrated with the experimental results (Section 2). The contribution of major scattering mechanisms for bulk materials is evaluated in Section 3.1. For deca-nanometer linewidth interconnects, the influence of GBS and SRS on resistivity is investigated, and the scaling effect is also elaborated in Section 3.2. Our results demonstrate GBS is the major mechanism of the scaling effect, and grain size has a significant influence on resistivity. Section 3.3 is devoted to evaluating the BPR materials with different aspect ratios. SRS is demonstrated to be the dominating mechanism for the resistivity drop in high-aspect-ratio rails.

## 2. Simulation Method and Scattering Mechanisms

Solving the BTE without using the relaxation time approximation [27,28] is a tough job. There are mainly two technical genres to solve it, namely deterministic methods [31,32,33] and the Monte Carlo approach [34,35]. Solving the BTE deterministically is advantageous to implement the Schrödinger equation (SE), Poisson equation (PE), and BTE self-consistent iterations in semiconductor device simulations; however, it has not been applied to the interconnect for the following reasons: (a) the SE-PE-BTE self-consistent iterative solver is very time-consuming and program-extensive; (b) the linewidth of interconnects is usually one order of magnitude larger than the channel thickness in FinFETs or ultra-thin-body devices, and, therefore, the quantum confinement effect in metallic lines is less conspicuous for the linewidth thicker than 6–8 monolayers [36]. For a typical linewidth of deca-nanometer scale, the Monte Carlo approach is the ideal solution to solve the BTE.

In our Monte Carlo method, the electron transport process is divided into free flight and scattering mechanisms, which occur alternatively. Numerous artificial particles, which act like electrons in the semiclassical frame, are planted into the simulated structure to experience the free flight and scattering processes. Stochastic numbers are used to determine which process or scattering event will happen. After the convergence, all macroscopic quantities, such as density and current, can be generated statistically. Five major scattering mechanisms are considered in our MC simulator, including acoustic phonon scattering (APS), electron to electron scattering (EES), plasma excimer scattering (PES), grain boundary scattering, and surface roughness scattering. The maximum time step will be determined as 2 × 10^4^ to achieve a balance between time consumption and simulation accuracy. Each simulation task requires roughly 4 GB of memory and takes about 48 h at AMD (Santa Clara, CA, USA) Ryzen ^TM^ 4800H processors.

Considering the average electron energy is several orders of magnitude higher than that of acoustic phonons under high electric fields, APS can be regarded as an elastic process approximately with a scattering rate for both absorption and emission processes [34]:

(1) $$\lambda_{APS}=\frac{\sqrt{2}m^{\frac{3}{2}}\Xi^{2}k_{B}T}{\pi \hbar^{4}\rho v_{s}^{2}}\epsilon^{\frac{1}{2}}$$

where *m* is the effective mass, *Ξ* the acoustic deformation potential, *k_B_* the Boltzmann constant, *T* the lattice temperature, *ℏ* the reduced Planck constant, *ρ* the density, and *v_S_* the speed of sound in solids, and *ϵ* the initial energy. The effective mass of the four materials is extracted from the reported band structure, respectively [37,38,39,40].

Collisions between free electrons in metals redistribute their energy and momentum, but the total energy and momentum remain the same statistically. This Coulomb-potential-caused elastic scattering can be expressed as follows [41]:

(2) $$\lambda_{EES}=\frac{e^{2}mn}{4\sqrt{2}\pi \hbar \varepsilon_{h}N_{i}}\underset{k}{\sum }\frac{\left|k-k_{0}\right|}{\left(\left|k-k_{0}\right|+\beta^{2}\right)\beta^{2}}$$

where *n* is the electron concentration, *N_i_* the total number of the involved scattering electrons, *ε_h_* the high-frequency dielectric constant, ***k*** the final state wave vector, ***k*_0_** the initial wave vector, and *β* the reciprocal of Debye’s length.

Besides EES, the fluctuation of local electron concentration will cause a rapid change in the electron distribution proportionally. This fluctuation-induced scattering is referred to as plasma excimer scattering. For PES with the total number of the involved scattering electrons *N_i_* and fluctuation frequency *ω_p_*, the scattering rate can be expressed as:

(3) $$\lambda_{PES}=\frac{e^{2}m^{\frac{1}{2}}\omega_{p}}{4\sqrt{2}\pi \hbar \varepsilon_{h}\epsilon^{\frac{1}{2}}}\left(N_{i}+\frac{1}{2}\mp \frac{1}{2}\right)\ln\left|\frac{{\epsilon^{\prime }}^{\frac{1}{2}}+\epsilon^{\frac{1}{2}}}{{\epsilon^{\prime }}^{\frac{1}{2}}-\epsilon^{\frac{1}{2}}}\right|$$

Grain boundaries in conductive materials are becoming a major roadblock for electrons to transit through interconnects as the linewidth scales down to the electron mean-free-path region [7,25]. To simulate the grain boundary scattering in metal lines, a Sinc function is employed to reproduce the barrier potential, and, thus, the scattering rate can be written as:

(4) $$\lambda_{GBS}=\frac{\sqrt{2}m^{\frac{3}{2}}VP\left(sinc^{2}\left(x-x_{i}\right)\right)}{\pi \hbar^{4}}{\left(\epsilon \mp \hbar \omega_{g}\right)}^{-\frac{1}{2}}$$

where *P*, calibrated to 0.1 eV, is the barrier potential amplitude, *m* the effective mass, *V* the volume, and *x_i_* the position of the grain boundary. Here, we let $x_{i}=i\times a+\left(i-1\right)\times r$, where *r*, a random number between 10^−9^ and 10^−8^, represents the thickness of the grain boundary, and *a* the grain size. The adoption of the Sinc^2^ function rather than the *δ* function can avoid numerical issues.

Metal surface roughness has a strong influence on interconnect resistivity by changing the electron momentum during SRS [23]. In our program, a specular parameter [23,42] and a roughness coefficient [23,42] are employed to characterize the surface shape, and, thus, the scattering rate is:

(5) $$\lambda_{SRS}=\frac{2\pi m{\left(1-\mu \right)}^{2}\sigma^{2}}{\hbar^{3}}{\left(\frac{e^{2}}{\varepsilon_{h}}\right)}^{2}{\left(\frac{N_{s}}{2}\right)}^{2}\frac{1}{\pi }\int_{0}^{\pi }r\left(e^{\frac{q^{2}\Lambda^{2}}{4}}\right)\left(1-\cos\theta \right)d\theta$$

where *μ* is the specular parameter between irreflexive (*μ* = 0) and specular (*μ* = 1) surface scattering based on the Fuchs–Sondheimer model [23], *σ*^2^ the roughness coefficient of the surface, and *N_S_* the sheet electron density calculated from the density of states and Fermi–Dirac distribution function. In the electron energy-related integration core, *q = |**k’** − **k**|* is the wave vector difference between initial and final states, *r* a random number between 0.1 and 1, *θ* the scattering angle, and *Λ* the correlation length of surface roughness.

**Figure 1:**
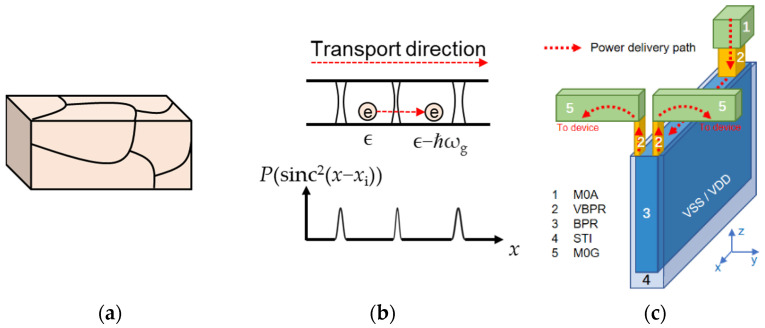
Schematic diagrams of simulated structures: (**a**) the simulated bulk-like structure and its grain boundaries, (**b**) the nanoscale interconnect grain boundaries and their Sinc function potential barriers, and (**c**) a schematic diagram of FS-BPR (front-side BPR) structure and power delivery path. In (**b**), an electron travels across a grain boundary and emits a phonon with energy ℏω_g_ to lattice. In (**c**), electric power distributes along 1. M0A (metal contact to active), 2. VBPR (via to the BPR), 3. BPR, and 5. M0G (metal contact to gate). A BPR is surrounded by STI and buried partially in STI oxide and Si substrate.

The APS, EES, and PES rates are bulk-like and are assumed to be linewidth-independent. Schematic diagrams of our simulated structure are shown in Figure 1. A phonon emission process caused by grain boundaries is illustrated in Figure 1b, as well as the potential of grain boundaries.

## 3. Results and Discussion

### 3.1. Analysis of Bulk Materials

**Figure 2:**
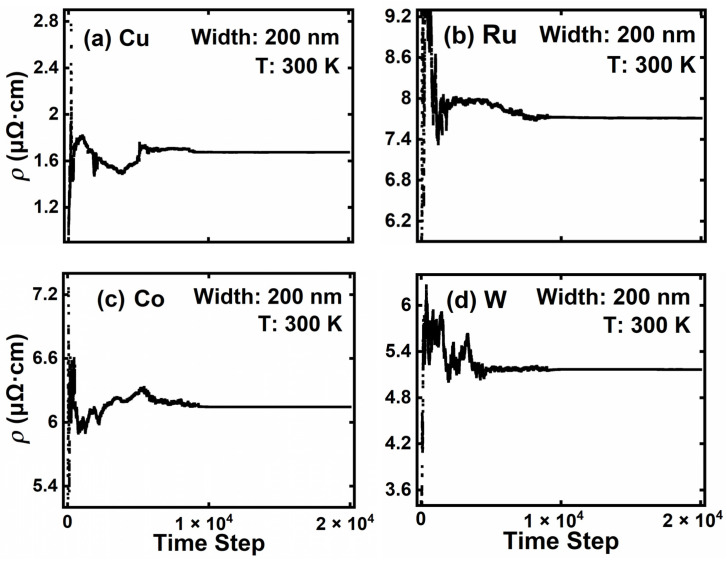
Bulk resistivity of (**a**) Cu, (**b**) Ru, (**c**) Co, and (**d**) W interconnect with the increase in time steps.

For the validation of our program and the calibration of the parameters, electron transport in bulk materials is simulated with the presence of APS, EES, and PES. In bulk materials, the grain size is approximately two orders of magnitude larger than the thickness of the grain boundaries [43,44]. Therefore, GBS can be ignored because the electron mean-free-path is much smaller than the typical grain size. In addition, interconnect linewidths are much larger than the electron mean-free-path in metals; therefore, the influence of SRS can be neglected. Figure 2 depicts the convergence behavior of the bulk average resistivity of the four metals at room temperature. The simulation of all four materials experiences drastic fluctuations in the first 6 × 10^3^ steps due to a poor initial guess of the electron distribution function, and then all converge steadily. A comparison between our simulation results and experimental bulk resistivities [45] is shown in Table 1. Good agreements are achieved for all four materials at room temperature with calibrated scattering parameters.

**Figure 3:**
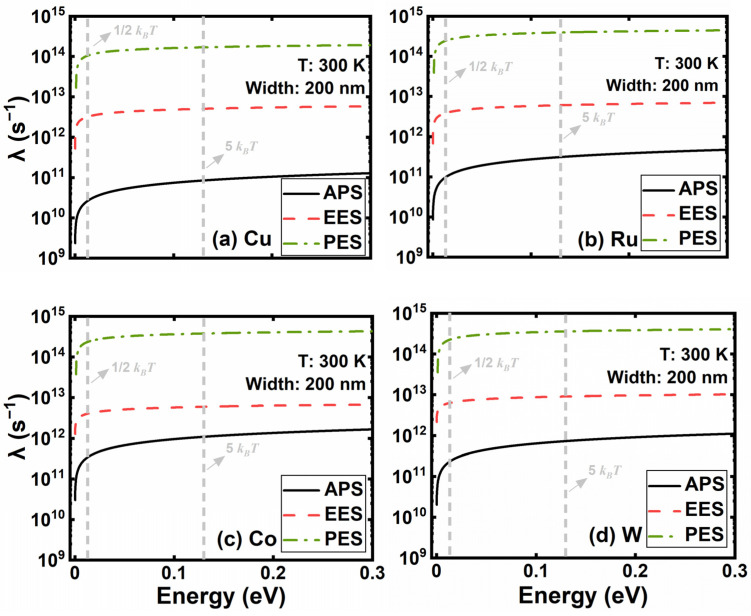
The scattering rates of bulk (**a**) Cu, (**b**) Ru, (**c**) Co, and (**d**) W with the electron energy ranging up to 0.3 eV.

To evaluate the impact of each scattering mechanism on electron transport, the scattering rate of electron energy ranging up to 0.3 eV is taken into account, as shown in Figure 3. As the electron energy increases from 0.013 eV, the scattering rate of EES and PES increase sharply first and almost remain the same at higher energy levels. Although the APS rate keeps increasing with energy, it is still at least an order of magnitude smaller than the other three. The scattering rate of PES is about 35 times larger than that of EES and plays a key role in bulk materials among the four scattering mechanisms.

### 3.2. Evaluation of the Scaling Effect

Electron scatterings at rough surfaces and grain boundaries are major causes of the resistivity increase in nanoscale interconnects. To evaluate the impacts of GBS and SRS, we further simulated a series of deca-nanometer metal lines, with their width varying from 48 nm down to 10 nm. According to the International Roadmap for Devices and Systems (IRDS) [46], the M0 layer should be at least 14 nm wide to preserve sufficient logic interconnect space for the middle end of line (MEOL). Hence, the minimum linewidth of the M0 layer is set to be 10 nm in this paper. The scattering rates of APS, EES, and PES are assumed to be linewidth-independent, while those of GBS and SRS are linewidth-related. Therefore, the scattering mechanisms for resistivity evaluation can be divided into these two cases.

**Figure 4:**
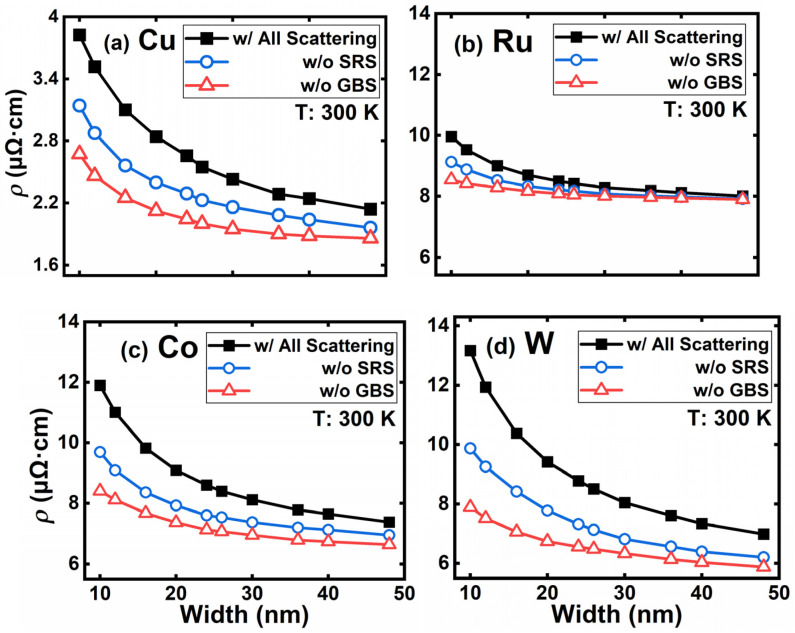
The resistivity of (**a**) Cu, (**b**) Ru, (**c**) Co, and (**d**) W interconnects with a linewidth ranging from 10 to 48 nm.

**Figure 5:**
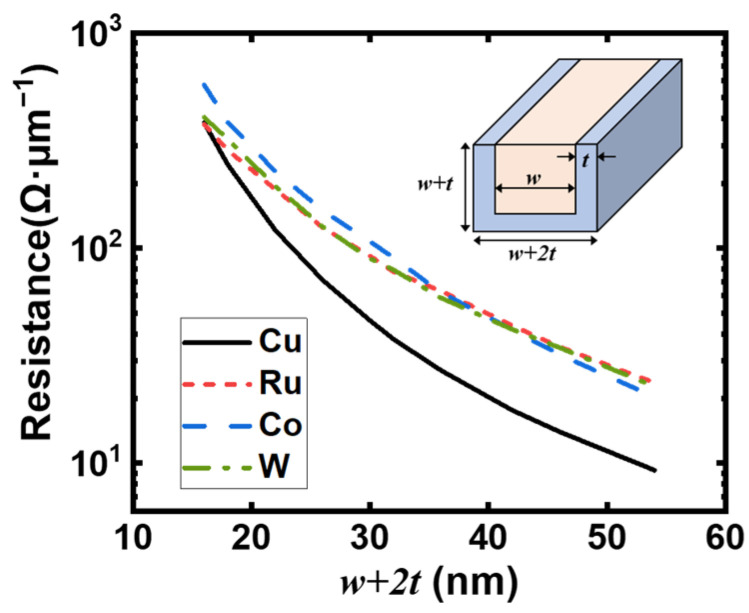
The calculated resistance per μm with liner thickness *t*^Cu^ = 3 nm, *t*^Ru^ = 0.3 nm, *t*^Co^ = 1 nm, and *t*^W^ = 0 nm. The inset demonstrates the structure of interconnect with the presence of liners and barriers. *w* is the width of the metallic lines, *t* the thickness of liner and barrier. All scattering mechanisms are included.

**Figure 6:**
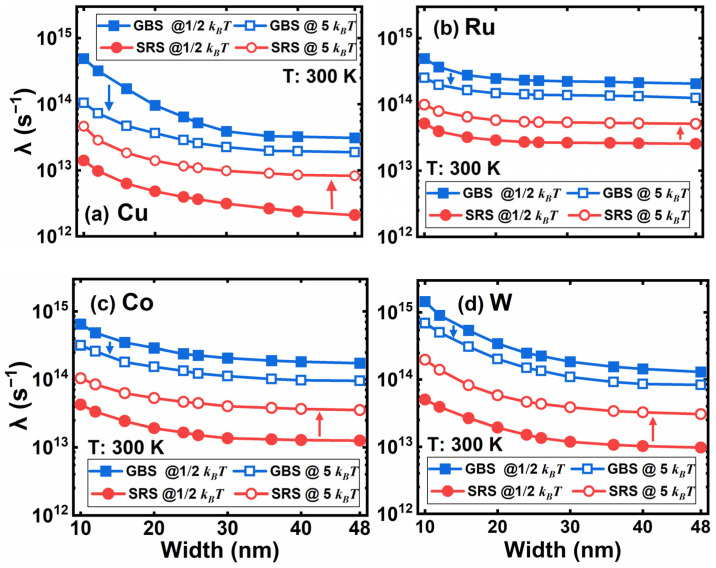
The scattering rate of GB and SR in (**a**) Cu, (**b**) Ru, (**c**) Co, and (**d**) W lines at electron energy levels of *k_B_T*/2 and 5 *k_B_T*. The linewidth varies from 48 down to 10 nm.

Assuming electrons mainly undergo diffusive scattering processes at interconnect surfaces, *μ*, *σ*^2^, and *Λ* in Equations (5) and (6) can be set to 0.2, 5, and 0.6. Figure 4 depicts the resistivity of each metal, with a linewidth ranging from 48 down to 10 nm at room temperature. The average grain sizes for simulations from Figure 4, Figure 5 and Figure 6 are listed in Table 2.

The cases without SRS or GBS are simulated in contrast to the case with all the scatterings to evaluate the impact of the two linewidth-dependent mechanisms. As the linewidth scales down, both GBS and SRS influence resistivity significantly. The absence of GBS results in much lower resistivities compared to the cases without SRS, suggesting GBS is the primary cause of the scaling effect, followed by SRS. For the case with all the scattering mechanisms, W shows the most dramatic resistivity rise and Ru the least. Although Cu shows the lowest resistivity at 10 nm, its scaling effect is one of the worst. Among the four materials, the scaling effect has the least impact on Ru.

The actual interconnects’ resistance depends not only on their resistivity but also on the volume occupied by the adhesion and wetting layers [47]. We hereby simulate the line resistance for the four materials with the presence of liners and barriers in Figure 5. Unlike the references [21,22] calculating the resistance analytically from the product of the bulk resistivity times the bulk electron mean-free-path, we implement all the scattering mechanisms inherently in the BTE by the Monte Carlo processes. The aspect ratio of the calculated interconnects is 1, and the state-of-the-art liner and barrier thickness is assumed for a fair comparison. The liner thickness for Cu, Ru, and Co are 3 nm, 0.3 nm, and 1 nm, respectively [48,49,50]. W has been reported for its linerless deposition, so no liner is added in this calculation [51]. For large linewidths, Cu still offers lower line resistance than its alternatives, as is expected due to its much lower resistivity and higher copper proportion in the total volume. However, with the decrease in linewidth (*w* + 2*t*), the copper proportion reduces rapidly and its resistance is finally analogous to the other three. As for Ru, Co, and W, although their resistivities show different trends with respect to linewidth, their resistance is surprisingly analogous to all the linewidths. Below about 20 nm, the superiority of Cu in resistance is significantly weakened, and anti-electromigration properties become a major concern in this region.

To further explore the origin of the scaling effect, we extracted the scattering rate of GBS and SRS with respect to electron energy in Figure 6. Considering most electrons are populated at lower energy states, the scattering rates of GBS and SRS at *k_B_T/*2 and 5 *k_B_T* are representative. In contrast to the four scattering mechanisms in Figure 3, the scattering rates of grain boundaries and surface roughness increase dramatically at lower energy levels and surpass those four scattering mechanisms at the energy level of *k_B_T* approximately.

It is worth noting that the GBS rate decreases with energy, and the SRS rate increases. The SRS rate is still one order of magnitude lower than the GBS rate at 5 *k_B_T*, where the electron distribution starts to be scarce. The reason is that electrons with higher energy are prone to be less affected by the potential energy of grain boundaries; however, the probability of a collision between hot electrons and the surface increases notably. Therefore, the scattering rate of GBS has larger magnitudes than SRS for the four metals and dominates the resistivity rises as linewidth scaling. As linewidth shrinks from 24 to 10 nm, the GBS and SRS rates at *k_B_T/*2 of W increase the most among the four materials, while the rates of Ru are much better, followed by Co. Consequently, in terms of the scaling effect, Ru is a good candidate for replacing Cu in M0 and M1, followed by Co.

**Figure 7:**
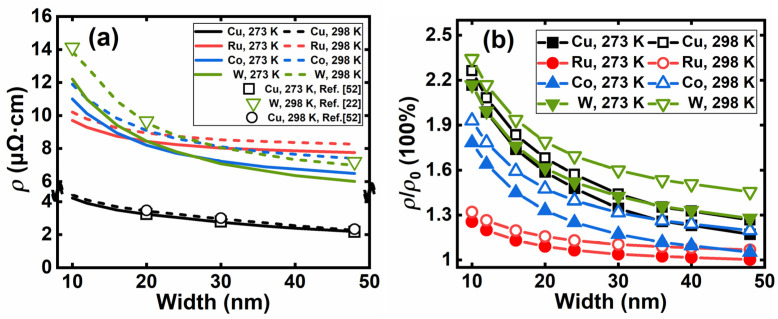
(**a**) Comparison of simulated resistivity and measured data at 273 and 298 K; (**b**) the ratio of the nanoscale interconnects resistivity *ρ* to bulk resistivity *ρ*_0_ as width scaling down at 273 and 298 K.

A comparison between the simulated resistivity and experimental data [22,52] at different temperatures is presented in Figure 7a. Good agreements between the simulation and available measured data at 273 K and 298 K are achieved, indicating our program is accurate for different metals at various temperatures at the deca-nanometer scale. Among the four materials, the temperature dependence of Cu is the least significant, followed by Ru. W is very sensitive to the change in temperature and linewidth.

Figure 7b illustrates the relationship between linewidth and *ρ/ρ*_0_ at different temperatures, where *ρ*_0_ is the bulk resistivity. W and Cu are both affected remarkably by scaling, while Ru is the least. Therefore, from the perspective of resistivity, Ru is a good alternative to Cu as a BEOL metallization material. However, the choice of M0, M1, and BPR metal is a complex compromise between resistivity, electromigration, contamination, etc.

**Figure 8:**
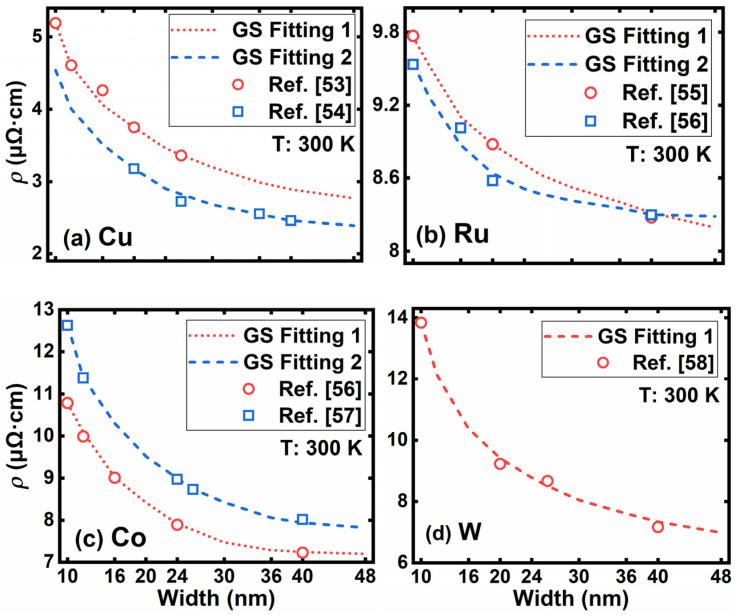
The fitting results of (**a**) Cu, (**b**) Ru, (**c**) Co, and (**d**) W with linewidth scaled from 48 nm to 10 nm. Hollow dots represent the experimental data, and the dashed lines are the simulation results with fitted grain sizes.

The grain size determines the behavior of GBS in nanocrystalline metals. In this work, the grain sizes are extracted from the experimental results [53,54,55,56,57,58]. The grain sizes of those reported epitaxial films are limited by their thickness, and the electron transport behaviors between electrodes are quasi-one-dimensional. Figure 8 presents the experimental resistivities and our simulation results with the corresponding fitted grain sizes of the four metals. The dashed lines are simulation results with the assumption of a full diffusive surface scattering (*μ* = 0). Very good agreements with the experimental data [36,37,38,39,40,41] are achieved for all four materials. The average grain sizes extracted from the fitted curves are listed in Table 3. Two sets of grain size (GS) curves for Cu, Ru, and Co correspond to two different process parameters, respectively.

### 3.3. Evaluation of BPR Materials

Ru and W are the two promising materials for BPR [8,9,10] for their high thermal budgets, relatively low resistance, and superior anti-electromigration properties. It has been reported that high-aspect-ratio (AR) Ru BPR demonstrates excellent resistivity reduction [8]. In this section, we will focus on the impact of AR from the perspective of resistivity and its physical mechanisms behind.

The electron transport behavior in BPR is slightly different from BEOL, particularly for SRS. A schematic diagram of FS-BPR is shown in Figure 1c. A remarkable feature of BPR is its high AR, which is designed to enable further scaling by burying under the transistors to replace the above MEOL, and to boost performance by reducing the resistance and I-R drop of the power rail. The grain size is usually determined by the shorter edge of the rail, and, thus, a higher AR cannot alleviate the adverse impact of GBS on resistivity. Meanwhile, considering the VBPRs connecting the BPR to M0A and M0G from the top surface, SRS occurs mainly at the top, upper-left, and upper-right STI-BPR surfaces. Therefore, the SRS rate in BPR can be modified as follows:

(6) $$\lambda_{SRS}=\frac{2\pi m{\left(1-\mu \right)}^{3}\sigma^{2}d}{\hbar^{3}n\sqrt{\left(1-r\right)L}}{\left(\frac{e^{2}}{\varepsilon_{h}}\right)}^{2}{\left(\frac{N_{s}}{2}\right)}^{2}\frac{1}{\pi }\int_{0}^{\pi }r\left(e^{\frac{q^{2}\Lambda^{2}}{4}}\right)\left(1-cos\theta \right)d\theta$$

where *n* is the aspect ratio, d the electron-mean-path of the studied material, and L the height of the BPR structure in the z-direction. In this paper, the electron mean-free-paths are chosen as 39.9 nm, 6.59 nm, 7.77 nm, and 11.2 nm for Cu, Ru, Co, and W, respectively [45].

**Figure 9:**
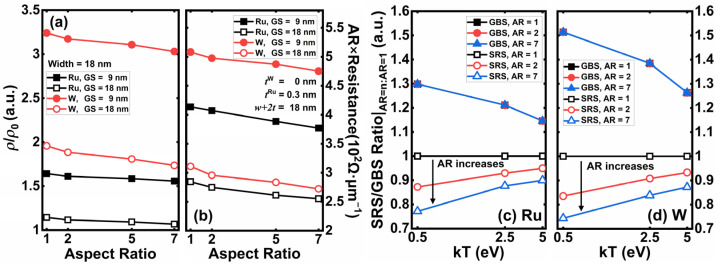
The impact of AR for Ru and W with a linewidth of 18 nm: (**a**) the ratio of the deca-nanoscale resistivity *ρ* to bulk resistivity *ρ*_0_ with GS = 9 and 18 nm; (**b**) the product of AR times resistance of Ru and W with GS = 9 and 18 nm; the scattering rate ratio of AR = *n* to AR = 1 as energy increases for (**c**) Ru and (**d**) W.

The resistivity of Ru and W with linewidth = 18 nm and ARs ranging from 1 to 7 are simulated in Figure 9. A bulk resistivity *ρ*_0_ is taken as a reference, and the ratio of simulated BPR resistivity *ρ* to *ρ*_0_ is extracted to evaluate the scaling effect of BPR in Figure 9a. Ru demonstrates a superior scaling nature at all ARs, even for GS = 9 nm. However, the resistivity of W with GS = 18 nm is almost two times larger than *ρ*_0_, and even exceeds three times for the case with GS = 9 nm. Grain size shows a significant influence on resistivity for both materials. In Figure 9b, the resistance of the two BPR candidates is calculated for different ARs. Considering the conductor volume is proportional to the AR with the same footprint (*w* + 2*t* = 18 nm), we use the AR times resistance product for a more intuitive comparison. The liner thickness of Ru is 0.3 nm, and W is linerless. The AR times resistance product benefits from the AR increase with both GS = 9 and 18 nm, which means the total BPR resistance may drop significantly. The key for a smaller BPR IR-drop is to keep its GS as large as possible.

Figure 9c,d shows the AR_GBS_ = *n*/AR_GBS_ = 1 and AR_SRS_ = *n*/AR_SRS_ = 1 (*n* = 1, 2, 7) for Ru and W in the energy space. As expected, the GBS rates are independent of AR for both Ru and W because GS is only determined by the short edge of the rail. However, the SRS rates are associated with the AR due to the shift in the surface area to volume ratio. A high AR reduces the SRS rates significantly at lower energy states, where the electron concentration is higher.

For high AR cases (e.g., AR = 7), the upper and lower surfaces are far enough apart and are separated by several grains. Electrons with lower energy can hardly cross *n* grains from one short edge and are affected by the opposite short edge’s SRS and, thus, behave like transporting in a three-surface rail. Only those electrons with rather high energy may have a chance to cross the vertical direction of the BPR and reach the opposite surface. In other words, the rail can be regarded as a four-surface conductor only for very hot electrons. That is the reason why AR_SRS_ = *n*/AR_SRS_ = 1 are both approximately 3/4 at low energy levels and converge to 1 as the energy increases for both Ru and W.

**Figure 10:**
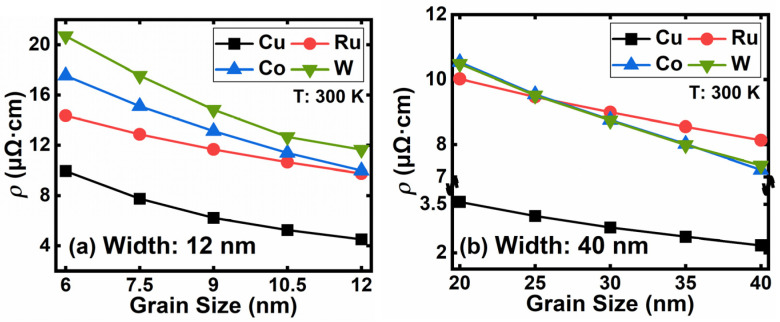
The resistivity of Cu, Ru, Co, and W at different GS with (**a**) 12 nm and (**b**) 40 nm linewidth.

To further evaluate the influence of GS on resistivity, we select 12 and 40 nm linewidth and vary the GS down to half-linewidth, respectively, in Figure 10. As the GS shrinks, the resistivity increases significantly, especially for smaller linewidth with smaller GS. With the decrease in GS, electrons face, accordingly, increased grain boundary barriers and lose energy during each GBS. Considering that the other scattering mechanisms (APS, EES, PES, and SRS) of Cu are relatively lower than those of Ru, W, and Co, the resistivity rise of Cu with a 12 nm linewidth and small GS are most notable. Among the four materials, the impact of GS on Ru is the least and W is the most, which is consistent with their scattering rate in Figure 6. Consequently, Ru is the most promising metal to replace Cu for further scaling. To achieve an ideal resistivity at future nodes, Ru metallization should be optimized for a larger GS and high AR.

## 4. Conclusions

The scaling effect of nanoscale MEOL and BPR interconnects was investigated by a self-developed BTE simulator based on the Monte Carlo approach. All the major scattering mechanisms, including APS, EES, PES, SRS, and GBS, were implemented in the simulator explicitly to capture the origin of resistivity of Cu, Ru, Co, and W. Good agreements of bulk resistivity between our calculations and experimental results were achieved. For nanoscale interconnects, GBS is the dominating mechanism of resistivity rises, followed by SRS and PES. The grain sizes of the reported experiments were extracted by our program for a better understanding of the grain-size-dependent resistivity for further scaling. The impact of AR was examined for the BPR application, and SRS was found to be the major cause of the resistivity decrease. The high-aspect-ratio SRS rates reduce by about 1/4 for the low energy states because high-AR rails act like a three-surface material for electron transport. The resistance with consideration of liners and barriers was simulated numerically instead of the previously reported analytical approximations. Ru, Co, and W demonstrate similar resistances at the deca-nanometer scale, whereas the Cu resistance is comparable to the other three below 20 nm linewidth due to its thicker liner requirements, although it exhibits much lower resistivity from bulk to nanoscale linewidth. Ru is the most promising MEOL and BPR metallization solution to replace Cu because of its better anti-electromigration properties, thinner liner requirement, and relatively lower resistivity. The key point to depressing the IR-drop of the power delivery network is to optimize the metallization process and AR to achieve larger grain sizes.

## Figures and Tables

**Table 1:** Comparison between simulation and experiment at 300 K.

Material	Simulation (μΩ·cm)	Experiment (μΩ·cm)
Cu	1.676	1.678
W	5.168	5.28
Co	6.145	6.2
Ru	7.71	7.8

**Table 2:** Average grain sizes for Cu, Ru, Co, and W for simulations from Figure 4, Figure 5 and Figure 6.

**Width (nm)**	10	12	16	20	24	26	36	40	48
**Average GS (nm)**	9.5	11.5	15	19	24	26	36	40	48

**Table 3:** Extracted grain sizes for Cu, Ru, Co, and W from experimental data.

Width (nm)	Grain Size (nm)						
	Cu^GS1^	Cu^GS2^	Ru^GS1^	Ru^GS2^	Co^GS1^	Co^GS2^	W^GS1^
10	8.7		8.3	9.1	10	8.7	8.8
12	10.3				12	10.5	
16	14.1			15.6	14.5		
20	17.9	18.6	18.5	19.7			18.8
24					22	19	
26	24	25				23	25.1
36		35					
40		38	37	37.5	40	35	38

## Data Availability

The data presented in this study are available on request from the corresponding authors.
